# A meta-analysis and systematic review of percutaneous catheter drainage in treating infected pancreatitis necrosis: Erratum

**DOI:** 10.1097/MD.0000000000014457

**Published:** 2019-02-08

In the article, “A meta-analysis and systematic review of percutaneous catheter drainage in treating infected pancreatitis necrosis”,^[1]^ which appeared in Volume 97 Issue 47 of *Medicine*, the authors uploaded the incorrect figures and table. The correct ones appear below ().

**Figure 1:**
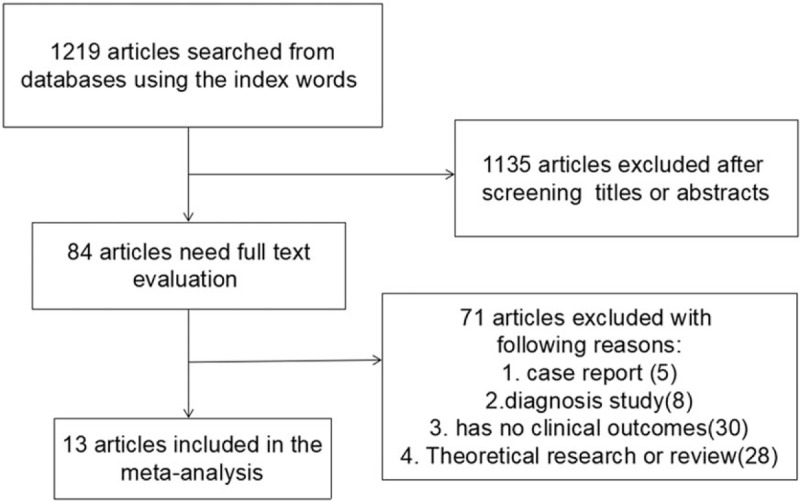
Flow diagram of the searching and selection process of literatures.

**Figure 2:**
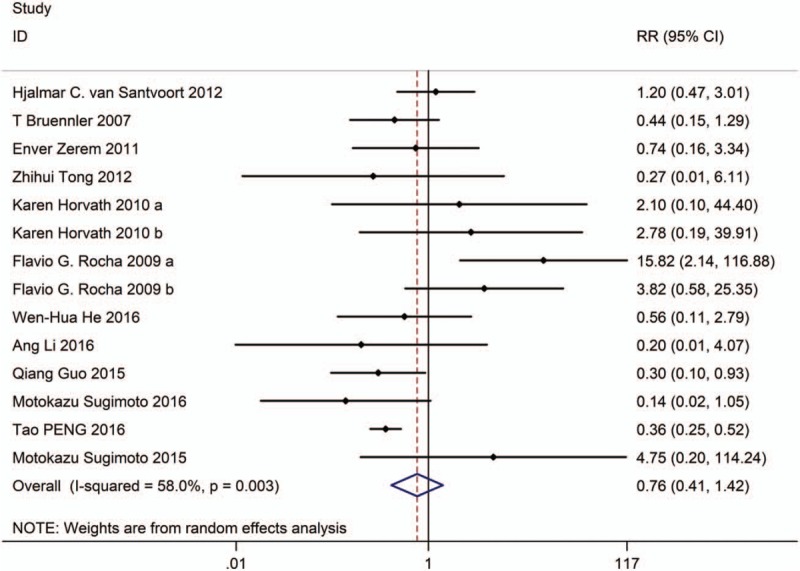
Forest plot showing the mortality of PCD versus surgical treatment.

**Figure 3:**
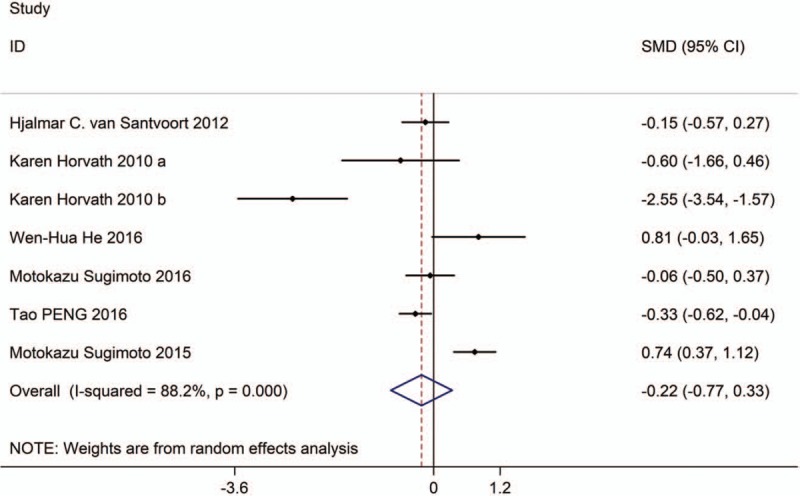
Forest plot showing the length of hospital stay of PCD versus surgical treatment.

**Figure 4:**
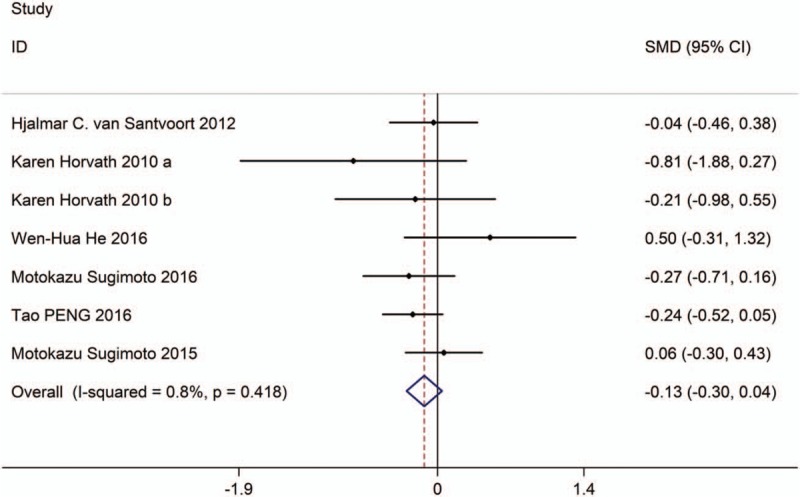
Forest plot showing the length of ICU stay of PCD versus surgical treatment.

**Figure 5:**
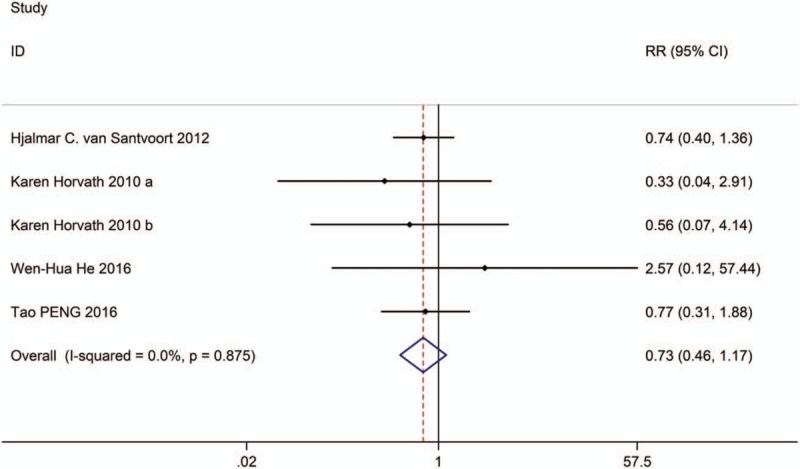
Forest plot showing the pancreatic fistula of PCD versus surgical treatment.

**Figure 6:**
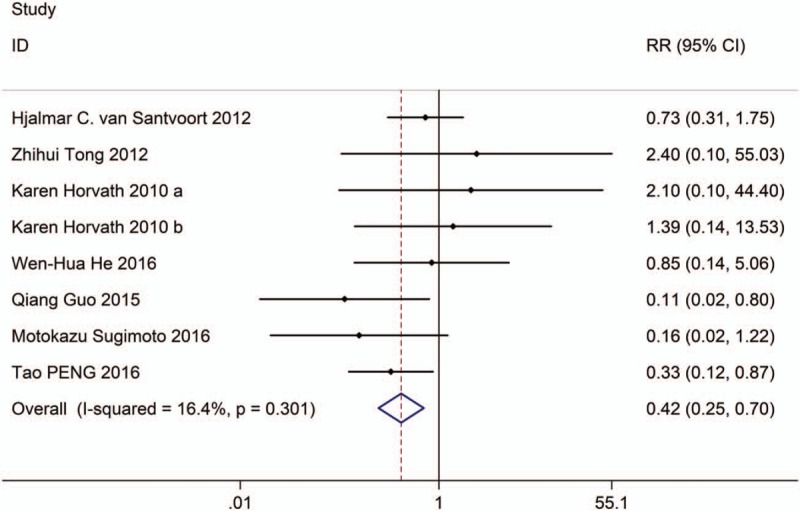
Forest plot showing the bleeding of PCD versus surgical treatment.

**Figure 7:**
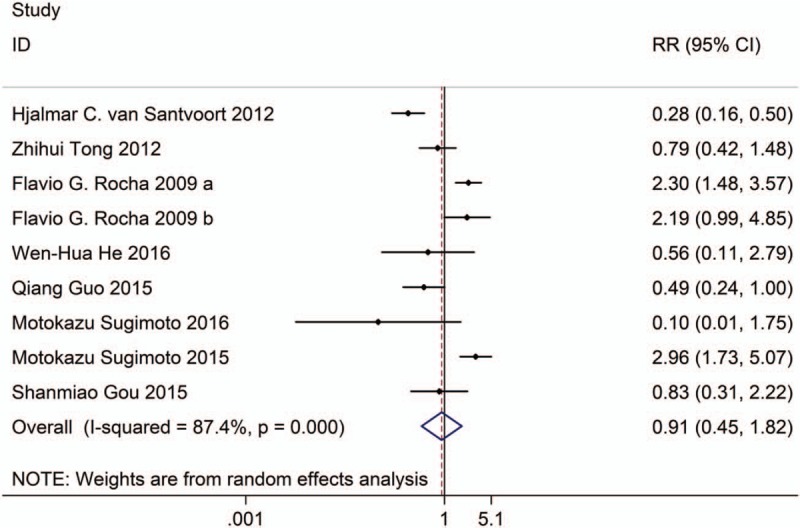
Forest plot showing the organ failure of PCD versus surgical treatment.

**Figure 8:**
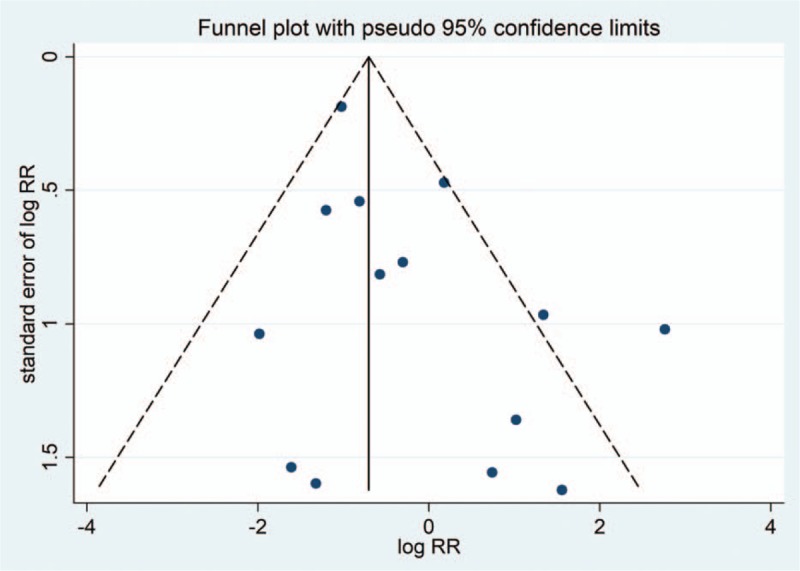
Funnel plot of studies in the meta-analysis.

**Table 1:**
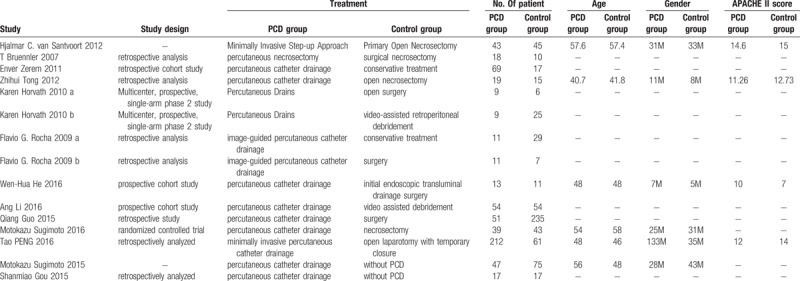
The major study characteristics.
